# Screening for *ELANE*, *HAX1* and *GFI1* gene mutations in children with neutropenia and clinical characterization of two novel mutations in *ELANE* gene

**DOI:** 10.1186/s12887-023-04428-w

**Published:** 2023-11-23

**Authors:** Patcharee Komvilaisak, Najwa Yudhasompop, Kittima Kanchanakamhaeng, Suradej Hongeng, Samart Pakakasama, Usanarat Anurathapan, Pongpak Pongphitcha, Duantida Songdej, Werasak Sasanakul, Nongnuch Sirachainan

**Affiliations:** 1 Departments of Pediatrics, Faculty of Medicine, Khon Kaen University, Khon Kaen, Thailand; 2 Department of Pediatrics, Hatyai Hospital, Hatyai, Songkhla Thailand; 3 Departments of Pediatrics, Sawanpracharak Hospital, Sawanpracharak Hospital, Nakhon Sawan, Thailand; 4 Department of Pediatrics, Faculty of Medicine Ramathibodi Hospital, Mahidol University, 270 Rama VI Road, Ratchathewi District, Bangkok, 10400 Thailand

**Keywords:** Severe congenital neutropenia, Cyclic neutropenia, *ELANE* Gene

## Abstract

**Background:**

Congenital neutropenia is a rare disease. Recurrent infections since young age are the presentation. The most common mutation causing severe congenital neutropenia (SCN) and cyclic neutropenia (CyN) is the *ELANE* gene. The objectives of this study were to screen the three common genetic mutations of *ELANE*, *HAX1* and *GFI1* in children with chronic neutropenia and to describe the clinical characteristics of children who had the mutations.

**Methods:**

Infants having ANC < 1,000/cu mm or children aged > 1 year having ANC < 1,500/cu mm at least 3 times in 3 months were enrolled in the study. Patients who had acquired neutropenia due to infection, immune deficiency, or drugs were excluded. The *ELANE* gene was first studied; and if mutations were not identified, the *HAX1* and *GFI1* genes were further examined.

**Results:**

A total of 60 patients were enrolled in the study. The median (range) age, ratio of female to male, ANC, and last follow-up age were 9.2 (0.5–45.2) months, 1:1.2, 248 (0–1,101) /cu mm, and 19.9 (3.5–202.3) months, respectively. Infections were noted in 67.3% of all patients. *ELANE* gene mutation was found in only four patients (6.7%), and the rest (56 patients) showed no mutations in the *HAX1* and *GFI1* genes. In patients without mutations, 66.0% had normal ANC during the follow-up, with a median (range) age for normal ANC of 19.8 (4.0–60.0) months. Two novel mutations p. Ala79del (c.234_236del) and p. Val197GlufsTer18 (c.589_590insAGGCCGGC) were identified, and they respectively cause SCN and CyN. Patients with the two novel mutations presented with several episodes of infection, including pneumonia, sepsis, abscess, otitis media, and gum infection.

**Conclusion:**

The genetic screening for *ELANE*, *HAX1*, and *GFI1* gene mutations in 60 patients with chronic neutropenia could identify four patients (6.7%) with *ELANE* gene mutation and two novel mutations, p. Ala79del in exon 3 and p. Val197GlufsTer18 in exon 4 causing SCN; and CyN, respectively.

## Introduction

Neutropenia is determined by the absolute neutrophil count (ANC) < 1,000/cu mm in the infant or < 1,500/cu mm in older children, which presents at least 3 times in 3 months. The etiologies of neutropenia are divided into congenital and acquired neutropenia. Congenital neutropenia is a rare disease with the prevalence of around 0.6-6/1,000,000 populations per year [1]. Congenital neutropenia includes Kostmann neutropenia, severe congenital neutropenia (SCN), cyclic neutropenia (CyN), and Shwachman-Diamond syndrome; while acquired neutropenia is caused by infection, drugs, nutrition deficiencies, and immune-related neutropenia [1]. Chronic idiopathic neutropenia (CIN) could be diagnosed by exclusion of other etiologies of neutropenia including autoimmune neutropenia (AIN). The most common presentation of neutropenia is a bacterial or fungal infection, especially when ANC is < 500/cu mm [2]. Laboratory diagnosis of neutropenia includes antineutrophil antibody detection, immunologic study, bone marrow aspiration (BMA), and DNA analysis. As the antineutrophil antibody test’s sensitivity and specificity were low (62.5% and 85%, respectively), a genetic study is helpful in suspicious patients with SCN or CyN [3].

A common genetic disorder of neutropenia is the *ELANE* or *ELA2*, which encodes neutrophil elastase. The gene is located on chromosome 19 (19p13.3) and consists of 5 exons, totaling 218 amino acids [3, 4]. Mutations of the *ELANE* gene are found in 40–50% of SCN, including CyN. Other than the *ELANE*, the *HAX1* gene, located on chromosome 1, has been reported in patients with Kostmann syndrome, delayed development, seizure monocytosis, and eosinophilia [5, 6]. Other rare genetic disorders of neutropenia are *GFI1*, *WAS*, *SDBS*, and *G6PC3* mutations. They usually have different manifestations, such as lymphopenia in *GFI1*, microcytic thrombocytopenia and immune deficiency in *WAS*, pancreatic insufficiency in *SDBS*, urogenital malformation, cardiac disorder, and myopathic syndrome in *G6PC3* gene mutations [2]. The objectives of this study were to screen the three common genetic mutations of *ELANE*, *HAX1* and *GFI1* in children with chronic neutropenia and to describe the clinical characteristics of children who had genetic mutations.

## Materials and methods

### Populations

After receiving informed consent from the parent, children under 15 years old having ANC < 1,000/cu mm in infants or < 1,500/cu mm in children aged > 1 year at least 3 times in 3 months were enrolled in the study. Patients who had acquired neutropenia due to infection, immune deficiency, or drugs were excluded. CyN was determined by the decrease in ANC with a 21-day cycle of at least 2 cycles [7]. This research was approved by the Ethics Committee of the Faculty of Medicine Ramathibodi Hospital (ID 02-56-44).

### Data and blood collection

Demographic data, including age, gender, presentation, laboratory findings, treatment, and outcome, were recorded. Three milliliters of blood were collected in an EDTA tube and centrifuged at 3,000 rpm to obtain a buffy coat. DNA was extracted by the standard technique. The *ELANE* gene was first studied due to the most common defect in children with congenital neutropenia. The sequencing study of *HAX1* and *GFI1* genes was followed if the *ELANE* gene study was negative.

### Analysis of ELANE, HAX1, and GFI1

*ELANE and HAX1* genes were analyzed according to the previously reported methods [4, 5]. For the *GFI1* gene located in chromosome 1 and having 7 exons, mutations have been reported only in exon 7 [5]. Our laboratory designed the primers as follows, 5’ AGGCCTTAGACTGTGGTG 3’ and 3’ GGGTCTGGAAAGTCAGAAG 5’. The PCR was carried out in 25 mcL of 100 ng genomic DNA, 10 pmol each primer, 0.2 mmol/L dNTPs, 5xGoTag Flexi buffer (Premega), 1.5 mmol/L magnesium chloride, and 0.5 U of GoTag Flexi DNA polymerase (Premega). The cycling profile consisted of the first step being held at 95^o^C for 5 min, followed by 30 cycles at 95^o^C for 45 s, 55^o^C for 1 min, and 72^o^C for 1 min. The PCR products were then submitted for sequencing. The variants were classified according to the ACMG classification criteria (including PM1, PM2, PM4, and BP4) [8].

## Results

A total of 60 patients were enrolled in the genetic study. The median (range) age, ratio of female to male, ANC, and last follow-up age were 9.2 (0.5–45.2) months, 1:1.2, 248 (0–1,101) /cu mm, and 19.9 (3.5–202.3) months, respectively. Infections were noted in 67.3% of all patients. *ELANE* gene mutation was found in only four patients (6.7%), and the rest (56 patients) showed no mutations in the *HAX1* and *GFI1* genes. The median (range) age at presentation of patients with *ELANE* gene mutations [3.6 (1.5–23.0) months] was younger than that of those without mutations [9.7 (0.5–45.2) months], but no statistical difference was identified, *P* = 0.22. In patients without mutations, 66.0% had normal ANC during the follow-up, with a median (range) age for normal ANC of 19.8 (4.0–60.0) months. The median time (95%CI) of follow-up to normal ANC was 14.2 (7.9–20.5) months.

Two of the four *ELANE* gene mutation patients, Patient 1 and Patient 2, had previously reported mutations p. Gly85Arg (c. 253G > A) causing SCN and p. Val101Met (c. 301G > A) causing CyN, respectively. Patient 1 (with p. Gly85Arg mutation) presented at the age of 1 month with severe *Pseudomonas aeruginosa* pneumonia and an ANC of 42/cu mm. He responded to a granulocyte colony forming unit (GCSF) of 10 mcg/kg/day, developed myelodysplastic syndrome at the age of 14 years old. The cytogenetic and next generation sequencing study revealed complex karyotypes of 46, XY, add(5)(q15)[13]/ 46, idem, del(4)(q21)[1]/ 46, XY, add(5)(q14)[21]/ 46, XY, add(5)(q13)[3]/ 46, XY[2], and mutations of *SETBP1* (c.2602G > A, p.D868N) and *NRAS* (c.34G > A, p.G12S) genes with an allelic fraction of 47% and 44%, respectively. The patient was well after 10/10 HLA matched hematopoietic stem cell transplantation. Patient 2 (with the p.Val101Met mutation) presented at the age of 1.5 months with sepsis and a low ANC of 180/cu mm. There were 21-day cycles of neutropenia observed before starting the GCSF. He responded to the GCSF at the dose of 13 mcg/kg/day, with the ANC maintained in the range of 500–1,000/cu mm. At the time of this report, he is 13 years old and doing well.

### A novel mutation (p. Ala79del) in *ELANE* gene causing severe congenital neutropenia

**Fig. 1:**
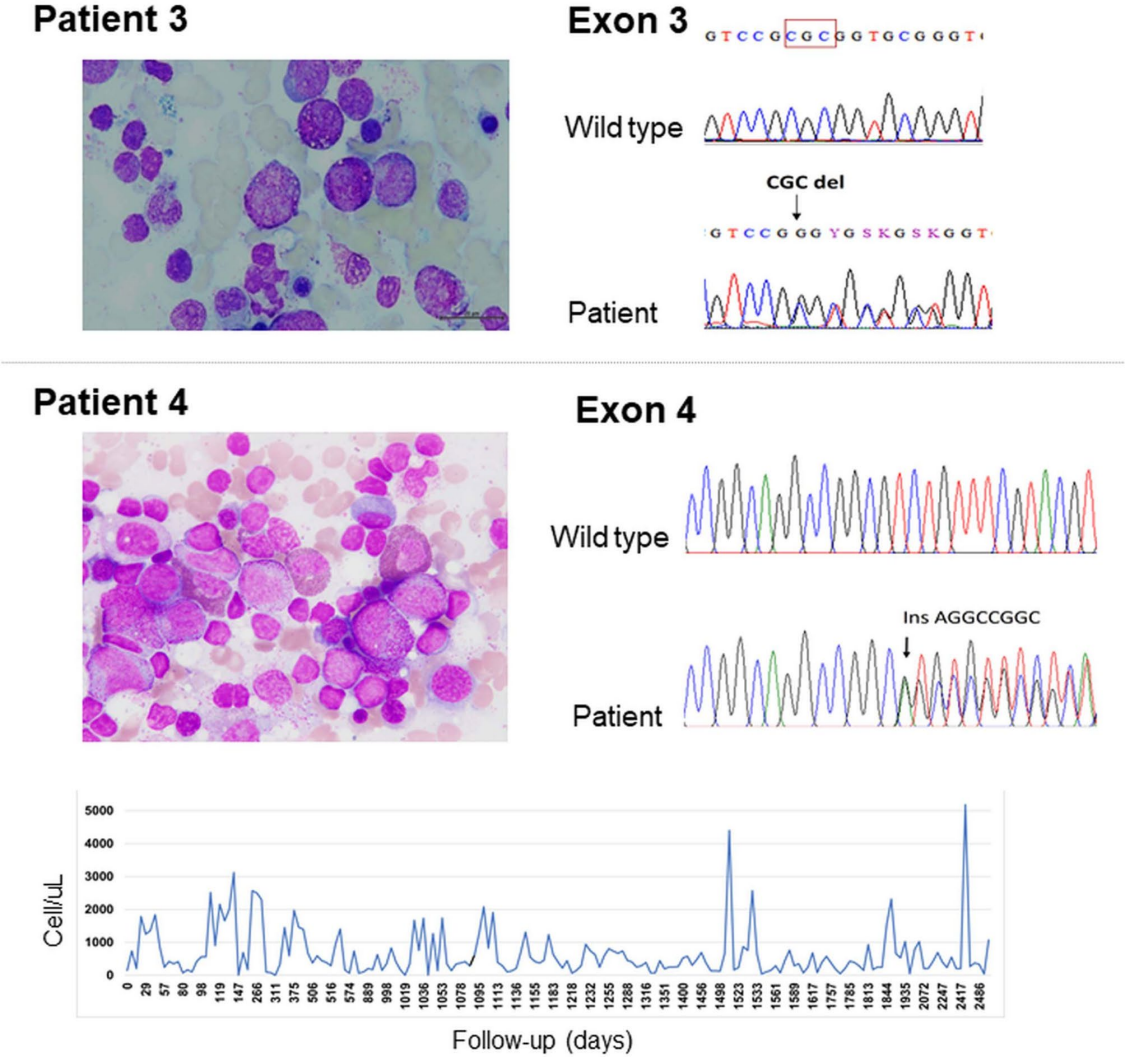
Bone marrow aspiration and chromatogram of Patient 3 and Patient 4 and absolute neutrophil counts in Patient 4

At the age of 21 days, Patient 3, presented with omphalitis. Her first complete blood count (CBC) showed normal hemoglobin (11.5 g/dL) and white blood cell (WBC) count (15,000/cu mm). However, the ANC was low at 300/cu mm (PMN 2%, lymphocyte 91%, monocyte 4%, and eosinophil 2%). She was treated and improved with a 7-day course of cloxacillin and cefotaxime. GCSF was given during the infection at 5 mcg/kg/day, and her ANCs were in the range of 480-1,190/ cu mm. One month later, she developed a fever with gingivitis and a seizure. Her meningeal sign was positive. The CBC showed mild anemia (hematocrit 25%) with a low ANC at 530/cu mm. BMA revealed maturation arrest at the promyelocyte and an increment of megakaryocytes with phagocytic activity. The cerebrospinal fluid examination showed a WBC of 3/cu mm and normal protein and sugar levels. Blood culture revealed Pseudomonas aeruginosa. She was then treated with a 3-week course of cefotaxime and amikacin. The GCSF was restarted at 10 mcg/kg/day, and the ANC responded at 2,500/cu mm. During the GSCF dose adjustment, she experienced three episodes of pneumonia within nine months. Finally, the patient had been on the GSCF at 5 mcg/kg/day. Her ANC was 1,336/cu mm. The genetic study of the *ELANE* gene revealed a novel mutation of c.234_236del, p. Ala79del, exon 3 (Fig. 1; Table 1). This variant was classified as a likely pathogenic variant according to the ACMG classification criteria, including PM1, PM2, PM4, PVS1, and BP4. At present, the patient is 6.1 years old and doing well without any infection.

**Table 1:** History and clinical characteristics of the four patients: Patients 1 and 2 were the previously reported mutations, Patients 3 and 4 were the novel mutations

	Patient 1	Patient 2	Patient 3	Patient 4
Gender	Male	Male	Female	Female
Age at presentation	1 month	1.5 months	21 days	1.9 years
Infection	Diarrhea, *Pseudomonas aeruginosa* pneumonia, Candida infection	Peritonitis, sepsis, pneumonia, skin, and liver abscess	Umbilical cord infection, gum infection, *Pseudomonas aeruginosa* sepsis, pneumonia	Pneumonia, cellulitis, abscesses, diarrhea, and otitis media
Initial ANC (/cu mm)	42	180	300	150
Clinical diagnosis	Severe congenital neutropenia	Cyclic neutropenia	Severe congenital neutropenia	Cyclic neutropenia
Bone marrow examination	Maturation arrest at promyelocyte	Maturation arrest at promyelocyte	Maturation arrest at promyelocyte	Maturation arrest at myelocyte
GCSF dosage (mcg/kg/day)	10	10	5	5.7
Other treatment	Haploidentical stem cell transplantation	None	None	None
FU time (year)	15.7	13.1	6.1	8
Outcome	Alive	Alive	Alive	Alive
Genetic study *ELANE* (NM_001972.4)	c.253G > A p.Gly85Arg, exon 3	c.301G > A p.Val101Met, exon 3	c.234_236delCGC p.Ala79del, exon 3	c.589_590insAGGCCGGC p.Val197GlufsTer18, exon 4
ACMG criteria	Previously reported mutation	Previously reported mutation	Likely pathogenic variant (PM1, PM4, PM2, PVS1, BP4)	Pathogenic variant (PVS1, PM2, PS2)
Parent(s)	Mother: normal	Mother and father: normal	ND	Mother and father: no mutation

ACMG, American College of Medical Genetics and Genomics; ND, Not done

### A novel mutation (p. Val197GlufsTer18) in *ELANE* gene causing cyclic neutropenia

At the age of 1.9 years, Patient 4 presented with persistent pneumonia in the left upper lobe and cervical lymphadenopathy. She began to have several episodes of fever from 5 months old to 1.9 years old, but the fever resolved without visiting a hospital. CBC revealed anemia (hemoglobin 7.5 g/dL), and normal WBC (7,380/cu mm) and platelet counts. ANC was low at 150/cu mm (PMN 2%, lymphocytes 80%, eosinophils 3%, monocytes 14%, basophils 1%). A lymph node biopsy showed reactive hyperplasia. She was treated with a broad-spectrum antibiotic and anti-tuberculous medication for one year. During follow-up without GSCF, ANC fluctuated between the normal and the lowest numbers at each cycle (Fig. 1). Recurrent infections were documented three times per year, including pneumonia, cellulitis, abscesses, diarrhea, and otitis media. She received GCSF (7 mcg/kg/day, 4 days per week) without infection. ANC was between 200 and 1,000/cu mm. BMA demonstrated normal cellularity with a maturation arrest at the promyelocyte. The genetic study of the *ELANE* gene revealed c.589_590insAGGCCGGC p.Val197GlufsTer18, exon 4. This variant was classified as a pathogenic variant according to the ACMG classification criteria, including PVS1, PM2, and PS2. Both parents of the patient did not have *ELANE* gene mutations (Fig. 1; Table 1). Presently, the patient is 7.7 years old; and with GCSF increased to 5.7 mcg/kg/day, she is doing well and with no infection.

## Discussion

Congenital neutropenia is a rare disease but should be suspected in patients who have presented with recurrent infections and prolonged neutropenia since infancy [1–3]. However, it is first necessary to rule out other commonly acquired neutropenia, for example, infection, drug, and immune deficiency diseases. Patients neither have secondary causes nor genetic mutations; CIN should be suspected. Furthermore, the positive anti-neutrophil antibody suggests the diagnosis of AIN. The common age of CIN and AIN was reported at 0.7, usually less than 2 years old. The etiology of CIN was reported to be due to impaired myeloid proliferation and/or maturation. In this study, most of the patients were not tested for anti-neutrophil antibodies due to the unavailability of the test. Besides, antibody identification was difficult to perform and might require several blood tests [9, 10]. The patients without genetic mutation in this report were suspected of having CIN or AIN; most of them (66%) recovered from neutropenia with a median (range) age for normal ANC of 19.8 (4.0–60.0) months, which corresponded to previously reported ages of around 3–5 years [11].

After screening of *ELANE*, *HAX1*, and *GFI1* genes in the enrolled patients, *ELANE* gene mutation was identified in 6.7% of patients. The *ELANE* gene encodes for neutrophil elastase, a myeloid cell-specific serine protease produced during the promyelocytic differentiation of neutrophils. Pathogenicity is defined as an increase in apoptosis in developing neutrophils caused by the unfolded protein response [1–3]. Individuals having mutations in the *ELANE* gene might exhibit SCN or CyN, which is the most prevalent genetic mutation presented in 50% of patients. Besides, the mutation is inherited in an autosomal dominant manner. The synthetic inactive form of ELANE protein consists of 267 amino acids and contains signal peptide, pro-dipeptide, mature protease, and C-terminal pro-peptide. After protein modification, the active enzyme comprises only 218 amino acids [12]. The most often reported mutations were missense mutations, which were followed by truncated, splice site, and in-frame deletions [13].

While SCN and CyN can result from mutations in the *ELANE* gene, descriptions of the disorders’ distinct features have been made. The clinical phenotypes showed a higher proportion of mouth ulcers and a lower proportion of pneumonia in CyN when compared to the phenotypes in SCN. In addition, patients with CyN required a lower dose of GCSF than the SCN patients [7]. The mutations in the *ELANE* gene can present as either SCN or CyN. It was demonstrated that mutations causing SCN or CyN were more widely distributed in all exons and introns, but the distribution of mutations in CyN was more frequently identified in exons 4 and 5 [7]. Moreover, a higher frequency of single-base pair and frameshift mutations was reported in SCN when compared to CyN [7]. Nevertheless, the clinical phenotypes, types of infection, and locations and types of genetic mutations cannot be completely differentiated between the two diseases [14].

While the known *ELANE* gene mutations were identified in Patient 1 and Patient 2, two novel mutations were each identified in Patient 3 and Patient 4. Patient 3 revealed the clinical characteristic of SCN, whereas Patient 4 revealed the clinical characteristic of CyN by displaying the oscillating pattern of neutrophil counts. Both patients had the same presentation with recurrent pneumonia and several sites of infection, i.e., omphalitis in Patient 3 and cellulitis and abscess in Patient 4. The most reported pathogen was *Staphylococcus aureus*, followed by *Escherichia coli* and *Pseudomonas aeruginosa*, respectively [15]. *Pseudomonas aeruginosa* was the identified pathogen in Patient 3; and no pathogen was identified in Patient 4. However, those abscess and cellulitis were common for *Staphylococcus aureus* infection; whereas diarrhea in Patient 4 could be due to *Escherichia coli*, a common pathogen in the gastrointestinal tract. After starting GCSF, the infections subsided. The average doses of GCSF in Patient 3 and Patient 4 were similar (5 mcg/kg/day and 5.7 mcg/kg/day, respectively), although a lower dose of GCSF was reported in CyN [7]. The mutations of c.234_236del, p. Ala79del in exon 3 in Patient 3 and c.589_590insAGGCCGGC, p. Val197GlufsTer18 in exon 4 in Patient 4 were identified. There was a study reporting similar locations to our report but different type of mutations of Ala79fs and Val197fs and showing characteristics of SCN [8]. Patient 4, who had p. Val197GlufsTer18 mutation, showed a distinct phenotype of CyN. The ACMG classification demonstrated likely pathogenic in Patient 3 and pathogenic in Patient 4 [8]. The pathogenesis of those mutations at exons 3 and 4 were predicted to disrupt the proposed transmembrane domain and disulfide bond domain in the C-terminus, respectively [4]. Without family histories of neutropenia in Patient 3 and no *ELANE* mutations identified in parents of Patient 4, sporadic mutations, commonly reported in *ELANE* gene mutation [16], were suspected in both patients.

Additionally, patients with *ELANE* mutations have an increased risk for MDS/AML. The leukemic transformation was reported by 20 years of age in 15–25% of the patients [15] and the reported mutations were p.Gly85Glu, p.Cys151Tyr, p.Gly214Arg, and frameshift mutations [7]. The presence of clonal mutations in monosomy 7 and 21, trisomy, chromosomal deletion, *CSF3R* mutations, and *RUNX1* mutations was demonstrated in [17, 18]. Patient 1, presenting the p. Gly85Arg mutation, developed MDS/AML at the age of 12.9 years. The clonal mutations in this patient consisted of *NRAS* (p.G12S) and *SETBP1* (p.D868N), instead of commonly reported *CSF3R* or *RUNX1* gene mutations. Those *NRAS* and *SETBP1* somatic mutations were demonstrated in MDS/AML [19, 20]. Because of the high prevalence of MDS/AML in SCN or CyN, especially when the age group approaches adolescence or young adulthood, patients should be screened for somatic mutations of MDS/AML.

In summary, the genetic screening for *ELANE*, *HAX1*, and *GFI1* gene mutations in 60 patients with chronic neutropenia could identify four patients (6.7%) with *ELANE* gene mutation and two novel mutations, p. Ala79del in exon 3 and p. Val197GlufsTer18 in exon 4 causing SCN; and CyN, respectively.

## Data Availability

Data are available from the corresponding author upon request.

## Abbreviations

AIN: Autoimmune neutropenia
ANC: Absolute neutrophil count
AML: Acute myeloid leukemia
BMA: Bone marrow aspiration
CBC: Complete blood count
CIN: Chronic idiopathic neutropenia
CyN: Cyclic neutropenia
GCSF: Granulocyte colony forming unit
MDS: Myelodysplastic syndrome
SCN: Severe congenital neutropenia

## References

[1] Fioredda F, Calvillo M, Bonanomi S, Coliva T, Tucci F, Farruggia P (2011). Congenital and acquired neutropenia consensus guidelines on diagnosis from the Neutropenia Committee of the Marrow Failure Syndrome Group of the AIEOP (Associazione Italiana Emato-Oncologia Pediatrica). Pediatr Blood Cancer.

[2] Donadieu J, Fenneteau O, Beaupain B, Mahlaoui N, Chantelot CB (2011). Congenital neutropenia: diagnosis, molecular basis, and patient management. Orphanet J Rare Dis.

[3] Dale DC, Person RE, Bolyard AA, Aprikyan AG, Bos C, Bonilla MA, Boxer LA (2000). Mutations in the gene encoding neutrophil elastase in congenital and cyclic neutropenia. Blood.

[4] Berliner N, Horwitz M, Loughran TP. Jr. Congenital and acquired neutropenia. Hematol Am Soc Hematol Educ Program. 2004;63–79. 10.1182/asheducation-2004.1.63. 10.1182/asheducation-2004.1.63 15561677

[5] Xia J, Bolyard AA, Rodger E, Stein S, Aprikyan AA, Dale DC, Link DC (2009). Prevalence of mutations in ELANE, GFI1, HAX1, SBDS, WAS and G6PC3 in patients with severe congenital neutropenia. Br J Haematol.

[6] Dale DC, Bolyard AA, Aprikyan A (2002). Cyclic neutropenia. Semin Hematol.

[7] Makaryan V, Zeidler C, Bolyard AA, Skokowa J, Rodger E, Kelley ML (2015). The diversity of mutations and clinical outcomes for ELANE-associated neutropenia. Curr Opin Hematol.

[8] Nykamp K, Anderson M, Powers M, Garcia J, Herrera B, Ho YY (2017). Sherloc: a comprehensive refinement of the ACMG-AMP variant classification criteria. Genet Med.

[9] Boxer L, Dale DC (2002). Neutropenia: causes and consequences. Semin Hematol.

[10] Walkovich K, Boxer LA (2013). How to approach neutropenia in childhood. Pediatr Rev.

[11] Dale DC, Bolyard AA (2017). An update on the diagnosis and treatment of chronic idiopathic neutropenia. Curr Opin Hematol.

[12] Rydzynska Z, Pawlik B, Krzyzanowski D, Mlynarski W, Madzio J (2021). Neutrophil elastase defects in congenital Neutropenia. Front Immunol.

[13] Rotulo GA, Plat G, Beaupain B, Blanche S, Moushous D, Sicre de Fontbrune F, Leblanc T, Renard C, Barlogis V, Vigue MG, Freycon C, Piguet C, Pasquet M, Fieschi C, Abou-Chahla W, Gandemer V, Rialland F, Millot F, Marie-Cardine A, Paillard C, Levy P, Aladjidi N, Biosse-Duplan M, Bellanné-Chantelot C, Donadieu J (2021). French severe chronic Neutropenia Registry. Recurrent bacterial Infections, but not fungal Infections, characterise patients with ELANE-related neutropenia: a French severe chronic Neutropenia Registry study. Br J Haematol.

[14] Horwitz MS, Corey SJ, Grimes HL, Tidwell T (2013). ELANE mutations in cyclic and severe congenital neutropenia: genetics and pathophysiology. Hematol Oncol Clin North Am.

[15] Rotulo GA, Beaupain B, Rialland F, Paillard C, Nachit O, Galambrun C, Gandemer V, Bertrand Y, Neven B, Dore E, Moshous D, Filhon B, Aladjdi N, de Sicre F, de la Tour RP, Ouachee M, Bellanne-Chantelot C, Dalle JH, Donadieu J (2020). HSCT may lower Leukemia risk in ELANE Neutropenia: a before-after study from the French severe congenital Neutropenia Registry. Bone Marrow Transplant.

[16] Ancliff PJ, Gale RE, Liesner R, Hann IM, Linch DC (2001). Mutations in the ELA2 gene encoding neutrophil elastase are present in most patients with sporadic severe congenital neutropenia but only in some patients with the familial form of the Disease. Blood.

[17] Freedman MH, Bonilla MA, Fier C, Bolyard AA, Scarlata D, Boxer LA, Brown S, Cham B, Kannourakis G, Kinsey SE, Mori PG, Cottle T, Welte K, Dale DC (2000). Myelodysplasia syndrome and acute Myeloid Leukemia in patients with congenital neutropenia receiving G-CSF therapy. Blood.

[18] Germeshausen M, Deerberg S, Peter Y, Reimer C, Kratz CP, Ballmaier M (2013). The spectrum of ELANE mutations and their implications in severe congenital and cyclic neutropenia. Hum Mutat.

[19] Tyner JW, Erickson H, Deininger MW, Willis SG, Eide CA, Levine RL, Heinrich MC, Gattermann N, Gilliland DG, Druker BJ, Loriaux MM (2009). High-throughput sequencing screen reveals novel, transforming RAS mutations in Myeloid Leukemia patients. Blood.

[20] Makishima H, Yoshida K, Nguyen N, Przychodzen B, Sanada M, Okuno Y, Ng KP, Gudmundsson KO, Vishwakarma BA, Jerez A, Gomez-Segui I, Takahashi M, Shiraishi Y, Nagata Y, Guinta K, Mori H, Sekeres MA, Chiba K, Tanaka H, Muramatsu H, Sakaguchi H, Paquette RL, McDevitt MA, Kojima S, Saunthararajah Y, Miyano S, Shih LY, Du Y, Ogawa S, Maciejewski JP (2013). Somatic SETBP1 mutations in myeloid malignancies. Nat Genet.

